# Reaction Characteristics of Andrographolide and its Analogue AL-1 with GSH, as a Simple Chemical Simulation of NF-κB Inhibition

**DOI:** 10.3390/molecules17010728

**Published:** 2012-01-12

**Authors:** Hui Yao, Sha Li, Pei Yu, Xiaodan Tang, Jie Jiang, Yuqiang Wang

**Affiliations:** 1 Department of Pharmaceutics, Jinan University College of Pharmacy, Guangzhou 510632, China; 2 Institute of New Drug Research, Jinan University College of Pharmacy, Guangzhou 510632, China; 3 Guangdong Province Key Laboratory of Pharmacodynamic Constituents of TCM, Jinan University College of Pharmacy, Guangzhou 510632, China

**Keywords:** andrographolide, andrographolide analogues, glutathione, NF-κB, medicinal chemistry

## Abstract

14-α-Lipoic acid-3,19-dihydroxyandrographolide (AL-1, **2**) is an analogue of andrographolide (Andro, **1**) coupled to α-lipoic acid (LA, **4**). AL-1 was at least 10-fold more potent than the natural parent compound Andro in inhibiting nuclear factor (NF)-κB activation in RIN-m cells. In the present study, glutathione (GSH, **3**) was used as a simple chemical model molecule of NF-κB with cysteine 62. The characteristics of the reaction between AL-1 or Andro and GSH were investigated to trace some possible elucidation for the inhibitive mechanism and stronger inhibition of AL-1 to NF-κB activation. The results showed that the main reaction products of AL-1 and Andro were identical, sulfhydryl adduct and amino adduct. AL-1 reacted much faster than Andro with GSH. The product yield of AL-1 was much higher than that of Andro. It was speculated that AL-1 might inhibit NF-κB by the same mechanism as Andro. And the faster reaction rate and higher yield may account for the stronger NF-κB inhibition of AL-1 when compared with Andro.

## 1. Introduction

**Figure 1:**
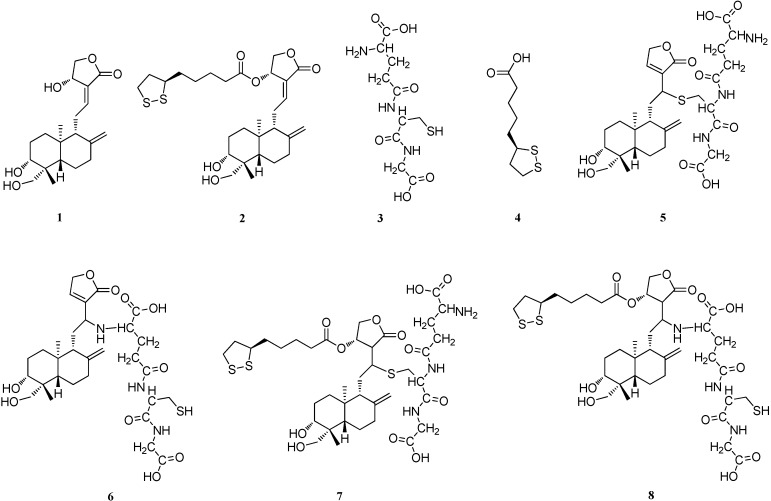
Structures of compounds **1–7**. Andro (**1**), AL-1 (**2**), GSH (**3**), LA (**4**), 14-deoxy-12-(glutathione-S-yl)-andrographolide (**5**), 14-deoxy-12-(glutathione-amino)-andrographo-lide (**6**), 12-(glutathione-S-yl)-14-lipoic acid-3,19-dihydroxyandrographolide (**7**), 12-(glutathione-amino)-14-lipoic acid-3,19-dihydroxyandrographolide (**8**).

Andrographolide (Andro), a diterpene lactone, is the major active ingredient in *Andrographis Paniculata* (Burm.f.) Nees. Extensive research revealed that Andro and its analogues have a broad range of beneficial pharmacological effects [1,2,3,4]. 14-α-Lipoic acid-3,19-dihydroxyandrographolide (AL-1, Figure 1) is an Andro analogue recently synthesized in our lab by esterifying the 14-hydroxyl group of Andro with α-lipoic acid (LA). AL-1 was more potent than Andro in various bioassays [5,6]. AL-1 showed potent activities closely related to the pathophysiological process of diabetes, including inhibition of nuclear factor (NF)-κB activation [7,8]. AL-1 was at least 10-fold more potent than Andro in inhibiting NF-κB activation induced by IL-1β and IFN-γ in RIN-m cells [7], an insulinoma cell line derived from a rat islet cell tumor. Although the precise mechanism of the action was still not completely understood, the strong inhibition of AL-1 to NF-κB activation may be one of the crucial reasons for its higher efficacy than Andro in diabetic animal models. However, the molecular mechanism underlining this inhibition is still unclear.

It was reported that Andro blocks the binding of NF-κB oligonucleotide to nuclear proteins, by forming a covalent adduct with the SH group of reduced cysteine 62 on the p50 subunit of NF-κB [9], thus inhibited NF-κB activation. Glutathione (GSH), a ubiquitous reducing sulfhydryl tripeptide, was well-known to be involved in many important cellular functions. Recently, studies demonstrated that the reduce of intracellular GSH level obviously augmented the cytotoxic activity of andrographolide to multiple myeloma cancer stem cells and human hepatoma cells respectively [10,11]. The action was thought to be partially resulted by the Andro’s binding to GSH [10,11,12]. Further study showed that the cysteine sulfhydryl group of GSH reacted with Andro to yield a sulfhydryl adduct [13]. Both GSH and NF-κB have a reduced cysteine with a sulfhydryl group, and both may interact with Andro to produce a sulfhydryl adduct. Therefore, in this work, we considered using GSH as a simple chemical model molecule of NF-κB with cysteine 62. By investigating the reaction between AL-1 and GSH through stoichiometric analysis, we aimed to find out whether the SH group of GSH reacts with AL-1, and whether there are differences between the reaction characteristics of AL-1 and those of its parent Andro. Thus, some information related to the probable molecular mechanism of AL-1’s inhibition of NF-κB activation and its higher inhibitive action compared with Andro may be obtained by this simple chemical simulation study.

## 2. Results and Discussion

### 2.1. Stability Analysis in the Absence of GSH

**Figure 2:**
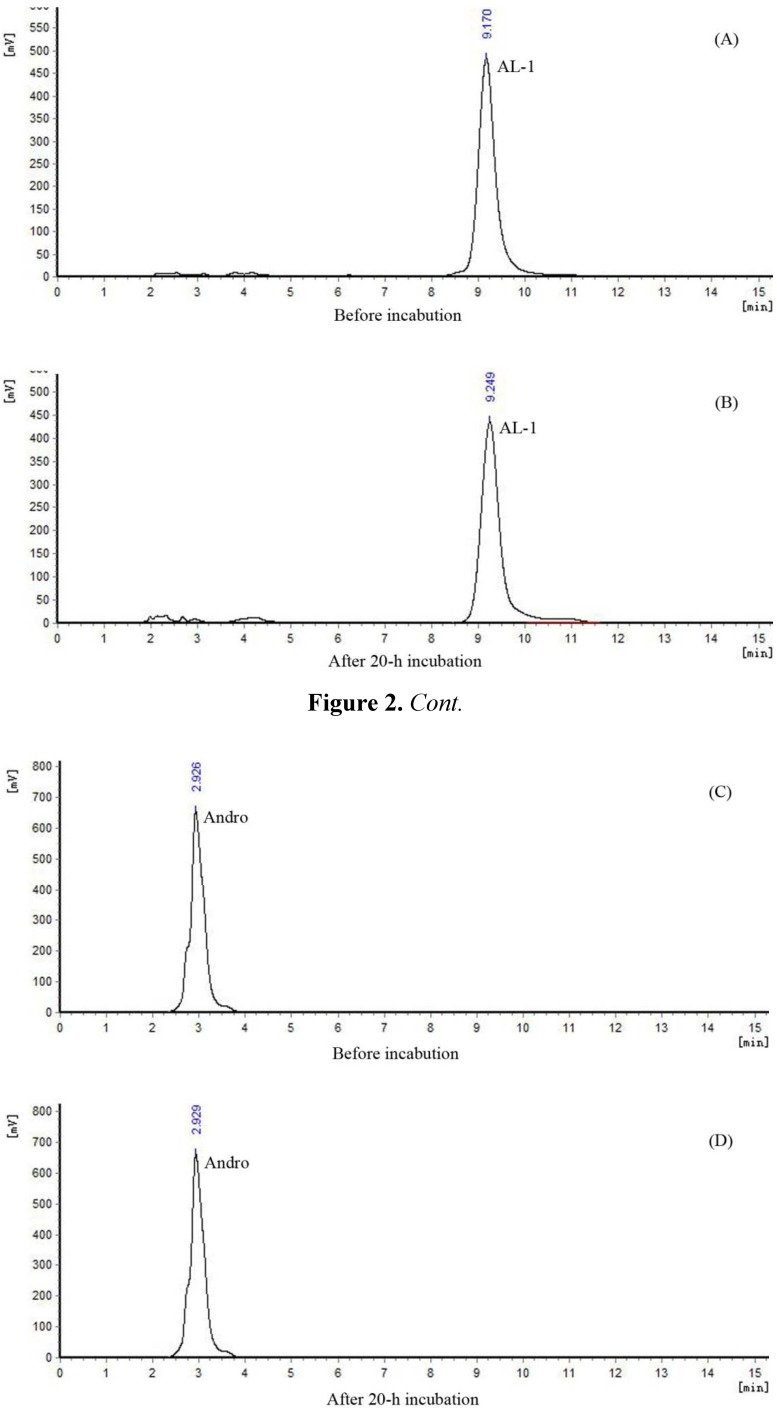
HPLC chromatograms of AL-1 and Andro in the absence of GSH.

Before studying the reaction between AL-1 and GSH, we first studied the stability of AL-1 in solution. Both AL-1 and Andro remained stable in the absence of GSH after 20 h of incubation at 37 °C in phosphate buffer solution (PBS, pH 7.4) (Figure 2A–D). The minor peaks in the chromatograms were not caused by reaction but resulted by the blank buffer solution. Therefore, all the minor peaks are ignored below.

### 2.2. Analysis of the Reaction Products between AL-1 and GSH

**Figure 3:**
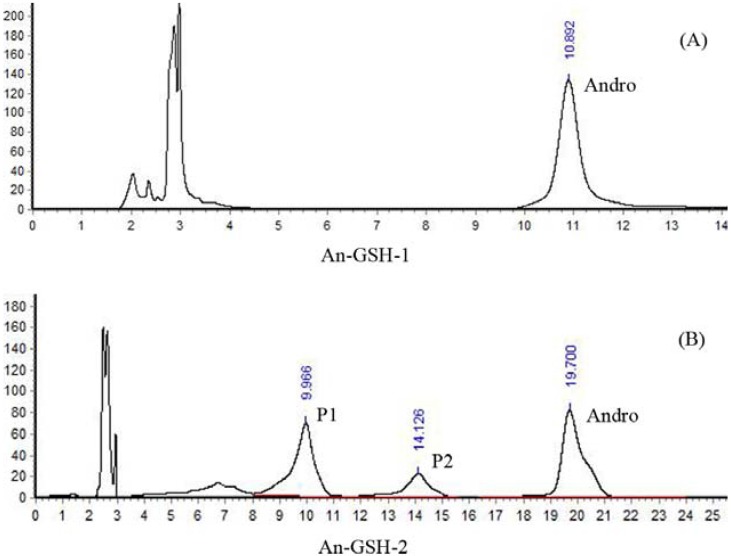
HPLC chromatograms of the reaction mixture of Andro and GSH. An-GSH-1 was analyzed by the mobile phase (1) methanol and water (52:48, v/v). An-GSH-2 was analyzed by the mobile phase (2) methanol and 0.1% v/v TFA solution (40:60, v/v).

**Figure 4:**
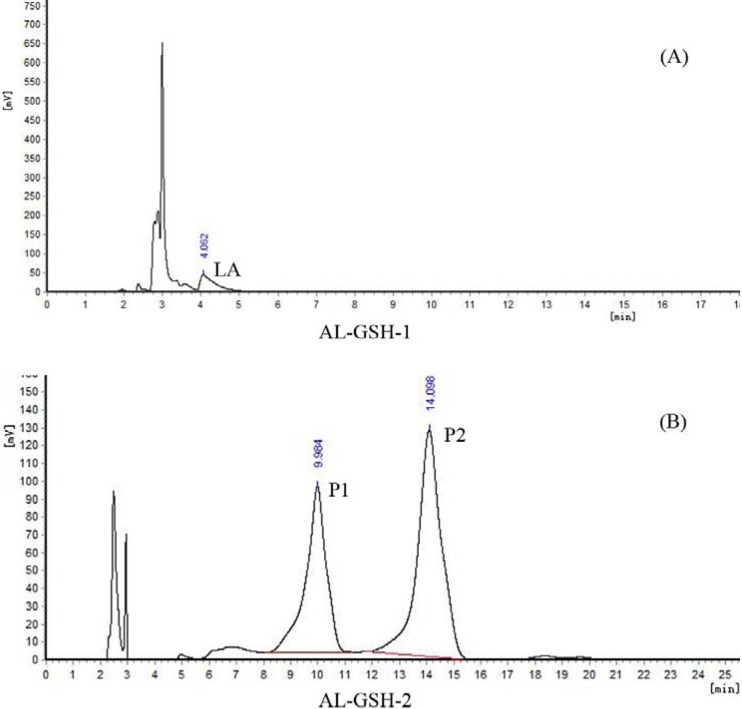
HPLC chromatograms of the reaction mixture of AL-1 and GSH. AL-GSH-1 was analyzed by the mobile phase (1) methanol and water (52:48, v/v). AL-GSH-2 was analyzed by the mobile phase (2) methanol and 0.1% v/v TFA solution (40:60, v/v).

In order to simulate the physiological environment, GSH was dissolved in PBS (pH 7.4). Ethanolic solutions of AL-1 and Andro were added to GSH solution, respectively, and then incubated overnight in a water bath at 37 °C, pH 7.4. The final concentration of all reactants was 1 mM. The reaction mixture of Andro and GSH (An-GSH) and that of AL-1 and GSH (AL-GSH) were analyzed by HPLC. We used two different mobile phases to identify the reactants and reaction products: (1) methanol and water (52:48, v/v) [14], and (2) methanol and 0.1% v/v trifluoroacetic acid (TFA) solution (40:60, v/v). The results are shown in Figure 3 and Figure 4.

After 20 h of incubation in the presence of GSH, the An-GSH reaction mixture was analyzed with the mobile phase (1), and there was still Andro left (Figure 3A). When it was analyzed using the mobile phase (2), two major products were found (Figure 3B). The one at about 10 min was termed as P1 and the other at 14 min was termed as P2. When the AL-GSH reaction mixture was analyzed with the mobile phase (1) after 20 h of incubation, both AL-1 and Andro was not found, but one product was identified as α-lipoic acid (LA) (Figure 4A). When it was analyzed using the mobile phase (2), surprisingly, the peaks at 10 min (P1) and 14 min (P2) were found again (Figure 4B).

**Figure 5:**
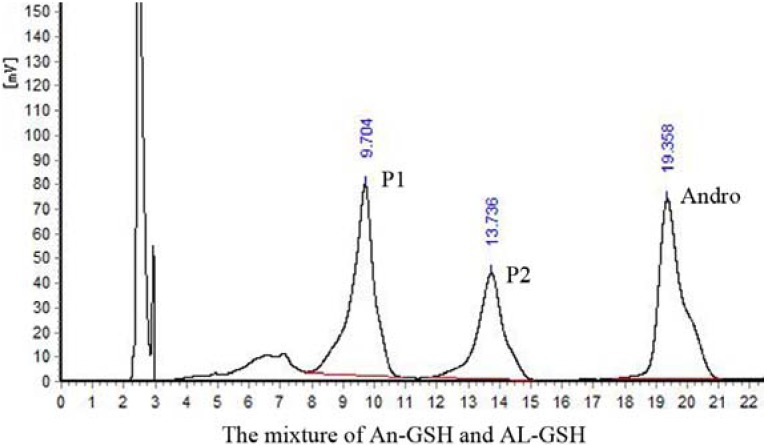
HPLC chromatogram of the mixture of An-GSH and AL-GSH reaction mixture. The mixture was analyzed by the mobile phase (2) methanol and 0.1% v/v TFA solution (40:60, v/v).

To confirm whether P1 and P2 in An-GSH and AL-GSH reaction mixture were the same, firstly, the reaction mixture of An-GSH and AL-GSH were mixed well and subjected to HPLC analysis. The results showed that the peaks of both P1 and P2 in An-GSH and in AL-GSH overlapped, indicating that they may be the same products (Figure 5).

### 2.3. Characterization of the Reaction Products

The reaction products were further characterized by both HPLC-ESI-MS and NMR. Two products of the reaction between Andro and GSH have been previously reported and characterized [13]. One was reported to be 14-deoxy-12-(glutathione-S-yl)-andrographolide (compound **5** in Figure 1), where the SH moiety of GSH reacted. The other is 14-deoxy-12-(glutathione-amino)-andrographolide (compound **6** in Figure 1), where the NH_2_ moiety of GSH reacted. For both the Andro and AL-1 reaction systems in this work, the HPLC-ESI-MS results showed that the quasi-molecular ion peaks of P1 were found to be *m/z* 640.8 [M+H]^+^ and 638.8 [M-H]^-^. Similarly, the quasi-molecular ion peaks of P2 were also found to be *m/z* 640.7 [M+H]^+^ and 638.8 [M−H]^−^. They were consistent with the theoretical molecular weight of the reported addition reaction products of Andro and GSH which is 639.8. Furthermore, P1 and P2 were isolated and purified by HPLC, and then subjected to NMR analysis. The NMR data were also found consistent with the above mentioned report [13]. P1 matched the data of compound **5** and P2 matched those of compound **6**. Therefore, in both reaction between AL-1 or Andro and GSH, two same products, sulfhydryl adduct (P1, compound **5**) and amino adduct (P2, compound **6**) were obtained.

### 2.4. Quantitative Monitoring of the Reactants and Products

**Figure 6:**
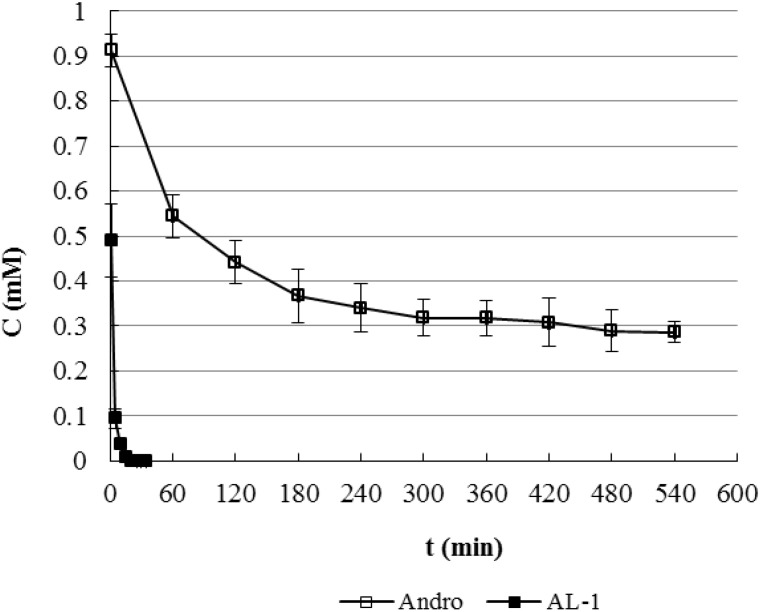
Change of Reactants in the Reactions of AL-1 and Andro with GSH (n = 3).

After finding that both the SH and NH_2_ moiety of GSH can react with AL-1 and Andro to yield P1 and P2 respectively, the reaction kinetics were investigated. First, calibration curves were constructed, respectively for AL-1 and Andro, in a gradient elution chromatographic condition, with the peak area plotted against the concentration of AL-1 and Andro ranging from 0.13 to 1.33 mM. Good linearity (R_AL-1_ = 0.9997, R_Andro_ = 0.9998) was obtained within this concentration range. The real-time monitoring of the disappearance of AL-1 and Andro was illustrated in Figure 6. AL-1 was found to disappear much faster than Andro in the presence of GSH. AL-1 completely reacted with GSH within 1 h. In contrast, only 37.7% of Andro reacted with GSH within the same time period. The half-life of AL-1 was only 2.1 min while Andro’s was 270.3 min.

**Figure 7:**
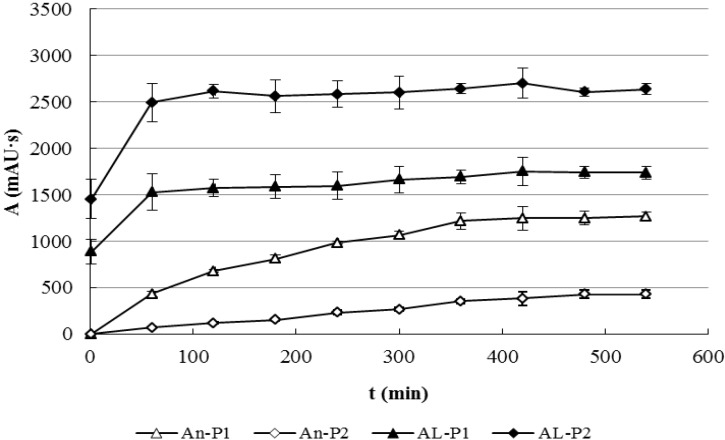
Comparison of Products in the Reactions of AL-1 and Andro with GSH (n = 3). An-P1, An-P2, AL-P1 and AL-P2 represented P1 and P2 in the Andro and AL-1 reaction system, respectively.

As the reaction proceeded, the concentration of Andro and AL-1 decreased and that of P1 and P2 increased (Figure 7). AL-1 not only reacted much faster than Andro, the concentration of P1 and P2 in the reaction of AL-1 with GSH were also much higher than that in the reaction of Andro with GSH. For example, after 120 min of reaction, the amount of P1 in the reaction of AL-1 with GSH was 2.3 times as much as that in the reaction of Andro with GSH; the amount of P2 in the reaction of AL-1 with GSH was 22 times as much as that in the reaction of Andro with GSH.

**Figure 8:**
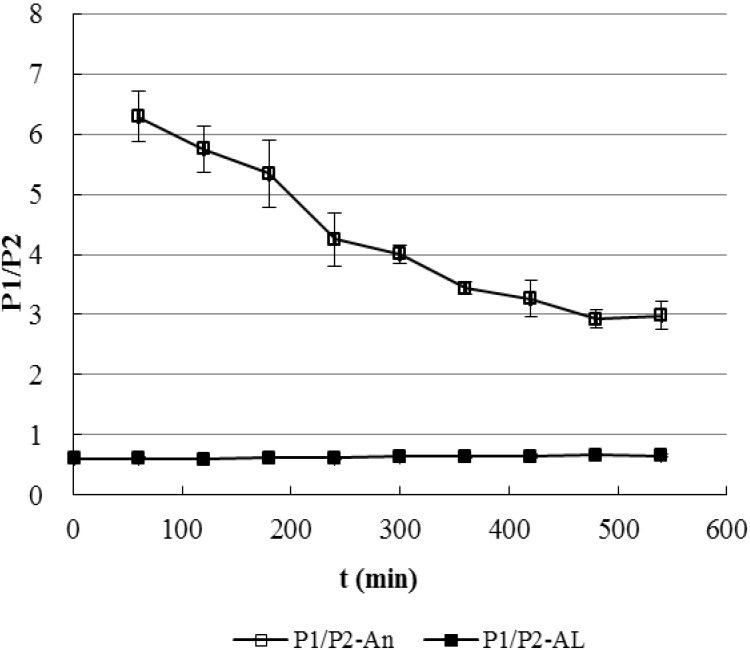
Real-time Monitoring of the peak area ratio of P1/P2 in the Reactions of Andro and AL-1 with GSH (n = 3). P1/P2-An and P1/P2-AL represented P1/P2 ratio in the Andro and AL-1 reaction system, respectively.

Finally, the peak area ratio of P1 to P2 (P1/P2) was plotted against time (Figure 8). The P1/P2 ratio of the AL-1 reaction system (0.60–0.67) was found quite different from that of the Andro reaction system (2.92–6.29) (Figure 8). Furthermore, the P1/P2 ratio in AL-1 system was much lower than that in Andro system at all times during the real-time monitoring.

### 2.5. Possible Reaction Mechanism

**Scheme 1:**
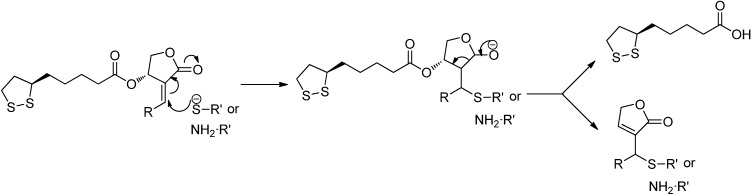
Possible Mechanism of the Reaction between AL-1 and GSH.

In the reaction of AL-1 with GSH, we initially speculated that the SH or NH_2_ group of GSH would directly add to the C12-C13 double bond to form two predicted products: 12-(glutathione-S-yl)-14-lipoic acid-3,19-dihydroxyandrographolide (compound **7** in Figure 1) and 12-(glutathione-amino)-14-lipoic acid-3,19-dihydroxyandrographolide (compound **8** in Figure 1). To our surprise, however, the two products from the reaction of AL-1 with GSH were the same as those from the reaction of Andro with GSH. The identical quasi-molecular ion peaks were observed by HPLC-ESI-MS for the two products of both reactions, and the same NMR spectra were also obtained. It has been reported that when Andro reacts with GSH, the α,β-unsaturated lactone moiety of Andro reacts with GSH through a Michael addition followed by dehydration of the adduct [13]. Since the main products of the reactions between AL-1 or Andro and GSH are the same, it is reasonable to assume that when AL-1 reacts with GSH, both the SH and NH_2_ moiety of GSH can add to the α,β-unsaturated lactone of AL-1, followed by the elimination of LA at C-14 to generate P1 or P2 (Scheme 1). We also observed the peak of LA in the chromatogram of the reaction mixture of AL-1 and GSH after overnight incubation (Figure 4A).

Due to the slow reaction rate and less steric hindrance, both SH and NH_2_ groups of GSH reacted at similar rate in the reaction between Andro and GSH. More sulfhydryl adduct P1 than amino adduct P2 (P1/P2 ratio >1) was produced possibly due to the higher stability of P1. The reaction may thus be a thermodynamically controlled process. However, the faster reaction of AL-1 with GSH may be a kinetically controlled process. Due to the fast reaction rate and higher steric hindrance, addition of the NH_2_ group of GSH may be favored over addition of the SH group because the SH group exists in the middle of the GSH molecule while the NH_2_ is a terminal group, making the SH group reaction sterically disfavored when attacking the double bond. This hypothesis explains why more P2 was yielded than P1 with a P1/P2 ratio less than 1, thus results in the different product ratio of this reaction compared to Andro’s.

Both the reactions of AL-1 and Andro with GSH yielded sulfhydryl adduct P1. AL-1 showed a faster reaction speed with a higher yield of sulfhydryl adduct. The yield of sulfhydryl adduct from AL-1 was about 1.4–3.5 folds as much as that from Andro in the 9-hour test. Within the first 60 min, the increasing rate of the peak area of sulfhydryl adduct in the AL-1 reaction system was 25.5 mAU·s/min while in Andro reaction system 7.2 mAU·s/min. Andro was reported to inhibit NF-κB activation by forming a covalent adduct with the SH group of the reduced cysteine 62 on the p50 subunit of the NF-κB [9]. In our pharmacological study, AL-1 was found to be significantly more potent in inhibiting NF-κB expression than Andro [7]. The results in this work suggested that AL-1 may inhibit NF-κB activation through the same mechanism as that proposed for Andro, *i.e.*, reacting with the SH group of cysteine 62 on the p50 subunit of the NF-κB. Like the reaction with GSH in buffered solution, AL-1 may react faster and yield a higher amount of sulfhydryl adduct than Andro in the interaction with NF-κB in cells, leading to a stronger inhibition of NF-κB expression.

## 3. Experimental

### 3.1. Chemicals

AL-1 was synthesized in our laboratory (>97%); Andro (>97%) was from Chengdu Okay Plant & Chemical Co. Ltd. (Sichuan, China); LA and TFA (≥99%) were purchased from Alfa Aesar (MA, USA); GSH (99%) was purchased from Kyowa Hakko Co. Ltd. (Japan).

### 3.2. HPLC Analysis

AL-1 or Andro solution (1 mM) were mixed well with GSH solution (1 mM) and then incubated in a water bath at 37 °C, pH 7.4. The reaction mixtures were sampled and analyzed directly by HPLC.

Gradient eluting was used for the real-time monitoring of the reactants and products of reaction. The mobile phase was solvent A (0.1% v/v TFA solution) and solvent B (methanol). The results of Figure 2,Figure 3,Figure 4,Figure 5 were assayed using a Shimadzu LC-10A system: Shimadzu LC-10AT vp and SPD-10A vp UV/vis detector (Shimadzu Co., Kyoto, Japan); column: Kromasil C18 (4.6 × 250 mm, 5 μm; Eka Chemicals, Bohus, Sweden). Figure 2: detection wavelength, 225 nm; mobile phase, methanol:acetonitrile:water = 70:10:20 (v/v). Figure 3A, Figure 4A: detection wavelength, 225 nm; mobile phase, methanol:water = 52:48 (v/v). Figure 3B, Figure 4B, Figure 5: detection wavelength, 210 nm; mobile phase, methanol: 0.1% TFA solution = 40:60 (v/v).

The results of Figure 6 and Figure 8 were assayed using an Agilent 1200 system: Agilent 1200 Series HPLC and UV-Vis detector (Agilent Technologies Inc., CA, USA); column: Kromasil C18 (4.6 × 250 mm, 5 μm; Eka Chemicals, Bohus, Sweden). Detection wavelength is 210 nm; and the gradient mode was: 0 min, 50% B; 70 min, 80% B.

### 3.3. HPLC-ESI-MS Conditions

AL-1 and GSH solutions were mixed and incubated overnight at 37 °C. The AL-GSH reaction mixture was extracted with CH_2_Cl_2_. The water phase was analyzed by HPLC-MS. HPLC-ESI-MS spectra were obtained using a Finnigan LCQ Advantage MAX mass spectrometer (Thermo Fisher Scientific Inc., MA, USA). Chromatograms were obtained from the Agilent 1200 Series HPLC with UV-Vis detector and Agilent ChemStation (Agilent Technologies Inc., CA, USA). Column: Agilent ZORBAX XDB-C18 (2.1 × 50 mm, 3.5 μm; Agilent Technologies Inc., CA, USA).

### 3.4. NMR

The NMR samples were obtained using a semi-preparation column Vydac (10 × 250 mm, 90 Å; The Separations Group Inc., CA, USA). ^1^H-NMR spectra were recorded at ambient temperature on an AVANCE III 300 MHz Digital NMR Spectrometer (Bruker, Karlsruhe, Germany).

### 3.5. *14-Deoxy-12-(glutathione-S-yl)-andrographolide (**5**)*

Amorphous substance. ^1^H-NMR (300 MHz, D_2_O) δ 0.55 (s, 3H, H-20), 0.94 (s, 3H, H-18), 0.96 (d, 1H, *J* = 13.2 Hz; H-5), 1.02 (overlapped, 2H, H-1), 1.08 (overlapped, 1H, H-5), 1.15 (overlapped, 4H, H-6, H-2), 1.17 (overlapped, 2H, H-11), 1.65 (overlapped, 1H, H-9), 1.74 (m, 1H, H-7), 1.82 (m, o, 2H, H-8′), 2.03 (m, 1H, H-7), 2.14 (m, 2H, H-7′), 2.74 (m, 1H, *J* = 14.4, 7.8 Hz, H-1′), 2.92 (m, 1H, *J* = 14.4, 7.8 Hz, H-1′), 3.31 (d, 1H, *J* = 11.1 Hz, H-19), 3.34 (m, o, 1H, H-3), 3.55 (overlapped, 1H, H-9′), 3.91 (overlapped, 2H, H-4′), 3.95 (overlapped, 1H, H-12), 4.00 (d, 1H, *J* = 11.4 Hz, H-19), 4.44 (dd, 1H, *J* = 7.8, 5.4 Hz, H-2′), 4.52 (s, 1H, H-17), 4.85 (s, 1H, H-17), 4.88 (s, 2H, H-15), 7.56 (s, 1H, H-14).

### 3.6. *14-Deoxy-12-(glutathione-amino)-andrographolide (**6**)*

Amorphous substance. ^1^H-NMR (300 MHz, D_2_O) δ 0.54 (s, 3H, H-20), 0.97 (m, 1H, H-1), 1.07 (s, 3H, H-18), 1.12 (d, 1H, *J* = 9 Hz, H-5), 1.15 (m, 1H, H-1), 1.20 (m, 1H, H-6), 1.69 (overlapped, 2H, H-2), 1.74 (overlapped, 2H, H-11), 1.85 (m, 1H, H-6), 1.89 (m, o, 1H, H-9), 1.98 (d, o, 1H, *J* = 13.8 Hz, H-7), 2.12 (m, o, 2H, H-8′), 2.17 (d, 1H, *J* = 12 Hz, H-7), 2.47 (m, 2H, H-7′), 2.65 (dd, 1H, *J* = 14.1, 8.7 Hz, H-1′), 2.82 (dd, 1H, *J* = 14.1, 5.7 Hz, H-1′), 3.30 (m, o, 1H, H-3), 3.38 (d, 1H, *J* = 12 Hz, H-19), 3.46 (overlapped, 1H, H-9′), 3.89 (overlapped, 2H, H-4′), 3.91 (overlapped, 1H, H-12), 3.96 (d, 1H, *J* = 11.4 Hz, H-19), 4.28 (dd, 1H, *J* = 8.7, 5.7 Hz, H-2′), 4.42 (s, 1H, H-17), 4.79 (s, 1H, H-17), 4.88 (s, 2H, H-15), 7.55 (s, 1H, H-14).

## 4. Conclusions

In this work, GSH was used as a simple chemical model molecule of NF-κB to simulate the possible interaction between an anologue of Andro, AL-1 and NF-κB. The reaction of AL-1 with GSH produced the two same products as those of Andro with GSH. However, AL-1 reacted much faster with GSH than Andro and produced more sulfhydryl adduct (P1). These results suggested that AL-1 might interact with NF-κB and share the same mechanism of Andro in NF-κB inhibition in cells, forming a covalent adduct with the SH group of the reduced cysteine 62 on the p50 subunit of NF-κB. AL-1 may interact more efficiently with the SH of NF-κB to afford higher protection to islet beta cells from damage *in vitro* and in diabetic animal models, when compared with Andro. Herein we just speculated about the interaction between AL-1 and NF-κB by simple chemical simulation. However, it still needs further exploration through biomedical research to elucidate the interaction mechanism and characteristics between AL-1 and NF-κB, as well as to verify our speculations in this work.
